# Effect on Perceived Weight of Object Shapes

**DOI:** 10.3390/ijerph19169877

**Published:** 2022-08-11

**Authors:** Taebeum Ryu, Jaehyun Park, Olga Vl. Bitkina

**Affiliations:** 1 Department of Industrial and Management Engineering, Hanbat National University, Daejeon 34158, Korea; 2 Department of Industrial and Management Engineering, Incheon National University, Incheon 22012, Korea

**Keywords:** shape, size, weight, perceived weight, size-weight illusion, user experience

## Abstract

The perceived weight of an object is an important research topic in terms of sensation and perception, and it is known that it has size-weight, color-weight, and material-weight illusions due to the influence of size, color, and material, as well as the weight of the object. Although the physical size of an object is measured by volume, the size of an object that we subjectively feel depends on the shape of the object, even if it has the same volume. Therefore, the shape of the object may determine the perceived size of the object, thereby changing its perceived weight accordingly. These cognitive factors play an important role in the period of rehabilitation therapy after an exacerbation or attack of neurological diseases, such as stroke or Parkinson’s disease, regarding the motor functions of the patient. Moreover, the study of these sensation and perception factors is important for the period of the early development of children, for example, for tracking and correcting fine motor skills. Existing related studies analyzed the perceived weight according to three shapes (tetrahedron, cube, and sphere), but only some shapes showed a difference in the perceived weight. This study attempted to demonstrate the difference in perceived weight according to the shape that has yet to be clearly identified. To this end, this study investigated objects with the same physical size (volume) as in previous studies, but in the shapes of tetrahedron, cube, and sphere. In addition, the volumes of these objects were set to 64,000 cm^3^, 125,000 cm^3^, and 216,000 cm^3^, and their weights were set to be 100 g, 150 g, and 200 g, in proportion to the size of the small, medium, and large volumes, respectively. Thirty-eight college students (21 males, 17 females) participated and the perceived weight of a given object compared to a reference object was evaluated according to the modulus method used for sensory size measurement. The analysis of the experimental data found that both weight (volume) and shape had significant effects on the perceived weight. The results support that the shape of objects also led to the size-weight illusion phenomenon. At the same weight (volume), the perceived weight of an object according to shape decreased significantly in the order of sphere, cube, and tetrahedron. At the same volume level, subjective size according to shape is small in the order of tetrahedron, cube, and sphere. The results of weight perception according to shape in this study showed that the subjective size of an object according to shape had an effect on perceived weight.

## 1. Introduction

The perceived weight of an object has been studied for a long time as part of the study of the sense of force in the field of experimental psychology. Although there are different opinions, it is known that the perception of force and weight is calculated by considering both the motor command initiated by the brain and the sensation felt by skin contact [1]. This is one of the important functions of muscles. At this time, it is known that the size of the object has an important influence [2]. If the size of an object is large, our brain commands us to apply a greater force than the actual required amount and, if the object is small in size, on the contrary, it commands us to apply a smaller force. Consequently, when objects with the same weight but different sizes are lifted, the perceived weights are affected by the size and the difference occurs. This is called the size-weight illusion (SWI).

Besides the known size, other factors can also affect the perceived weight of an object. On the same principle as SWI, the brain thinks, in advance, that a metal object will be heavier than a wooden object. In addition to materials [3,4,5], social factors, such as gender and age [6], color [7], and volume [8], can also make an illusion. For reference, there have been opposing opinions, such as the study that perception would be corrected by feedback during the lifting process [9] and the study that a similar effect occurred even when the field of view was blocked [10]. Of course, there are counterarguments to this. For example, although a perceptual rebalancing process exists, an illusion also remains because it emphasizes unexpected information by integrating previous expectations with current perceptual information [11].

The perceived weight of an object can also be affected by its shape. The perceived weight of an object is affected by its size and the factor that affects the perceived size is its shape. Dresslar made various flat shapes with lead and used it to study perceived weight, whereas Ellis used various types of solids with one weight (350 g) and volume (132 cm^3^) [12,13]. These two studies yielded contradictory results. The former reported the smallest-looking object as the heaviest, whereas the latter reported the largest-looking object as the heaviest. 

If the shape of an object affects the perception of size, a small object feels heavier than a large object of the same weight due to the SWI. Accordingly, there have been existing studies to find the relationship between shape and size-weight illusion. Kahrimanovic et al. [14] compared the subjective sizes of tetrahedron, cube, and sphere in the same volume and reported tetrahedron to be the largest in size, followed by the cube and the sphere. Based on the SWI phenomenon, the perceived weight of the sphere with the smallest sense of size at the same volume and weight would be the largest, followed by a cube and a tetrahedron. However, Kahrimanovic et al. [15] verified this prediction and found that the cube was statistically heavier than the tetrahedron and there was no difference in perceived weight between the sphere and the tetrahedron and between the sphere and the cube. The limitations of these two studies must be mentioned as they are similar to this study. In these studies, visual and tactile information was not used when the participants evaluated the perceived weight of an object during the experiment. Considering that in the real world, when we lift an object, the brain synthesizes visual, tactile, and other various feedback information, this is an important disadvantage.

The study of illusions is important for various fields and especially in medicine. Since any perceived illusions are associated with the work of different parts of the brain, their monitoring and study can help in the diagnosis and therapy of cognitive and neurological disorders. Research in [16] applied weight perception criteria for brain function study in sensory information processing for patients with autism. Research in [17] showed that size-weight illusion could be used for the assessment of visual–haptic integration in people with early visual deprivation. Moreover, the experiment with various geometric shapes used in the present study can also be used to diagnose and monitor the motor functions of the brain. The study of [16] showed that 3D virtual games with different geometric and toy shapes could be used for the development of fine motor skills in children.

The objective of this study was to analyze how the shape of an object mediates the SWI phenomenon. In order to achieve this research objective, the effect of the shape of tetrahedron, cube, and sphere on the perceived weight was investigated in this study. Although studies on the effect of the shape of an object on the perceived weight have been conducted, the research outputs have been contradictory or the theory explaining the effect of the shape of an object on the perceived weight has not been sufficiently explained experimentally. According to the predictions of previous studies, the perceived sizes of the three shapes increased in the order of sphere, cube, and tetrahedron, so their perceived weights increased in the same order. In this study, three shapes with the same weight and volume were created, with the volume set to three levels of small, medium, and large and the weight set to three levels by volume as well. Nine solids were used to have three-shaped solids for each volume level. This study will aid in revealing the effect of the shape of an object on the perceived weight. 

## 2. Background of Object Perception in the Fields of Cognition and Medicine

Previous research and medical experience show that after a stroke, an attack of Parkinson’s disease, as well as during the early development of children, including children with Down’s syndrome and cerebral palsy, the development and restoration of the body’s motor functions play a special role [18]. Motor functions are closely related to brain functions and one of the most effective medical approaches for the development and restoration of brain function is the performance of tasks related to fine motor skills in children and adults [19,20]. Fine motor skills tasks include drawing, writing, playing with small objects, and solving three-dimensional puzzles with different geometric shapes, such as sphere, cylinder, tetrahedron, and cube, in various weights, depending on the patient age and health issues. Further, working with these puzzles develops spatial and logical thinking, including the perception of the weight, forms, shapes, and colors of surrounding objects [18,21]. Based on these facts, for the presented study, the most common geometric shapes in rehabilitation tasks (sphere, tetrahedron, and cube) and various weights of figures (100–200 g) were selected to study cognitive functions in terms of object weight perception. The presented research direction was chosen based on previous publications demonstrating scientific results and evidence (Table 1). Note that in order to investigate the size-weight illusion phenomenon, a sphere, tetrahedron, cube, octahedron, and icosahedron, whose shape does not change significantly when viewed from various angles, were considered. Among those, octahedron and icosahedron were excluded in the current study for the efficient design of the experiment.

Based on the above studies (Table 1), research on perceived weight is not just basic science, it clearly shows that there is a possibility of practical application. Further, it was found that occupational therapy (including educational games with various geometric shapes) is one of the ways to restore and develop motor functions for people of different ages and health conditions [18,21,22]. Moreover, the connection of human motor functions, including fine motor skills, with cognitive functions, perceptual functions, and spatial thinking was also confirmed [17,20]. This is especially important for people with various neurological disorders and problems in early development. Since the illusion of perception of the weight, shape, density, and size of an object belongs to this category of medical problems, research in this area is also important in order to develop new, and improve existing, treatments for neurological disorders. The present article proposes new results on the relationship between human perception of size, weight, and shape of objects, which, in turn, can help find new methods for the development of young children, as well as recovery approaches from stroke, paralysis, and Parkinson’s disease.

## 3. Methods

### 3.1. Participants

The experiment in this study was approved by the Institutional Review Board (P01-201910-13-001). Thirty-eight healthy undergraduates in their 20s (21 males, 17 females) voluntarily participated in this experiment. They all passed the magnitude estimation test using the length and number of lines. This test is to check whether a participant has size estimation ability and followed the protocol used in a previous study [25,26]. It is known that more than 95% of the general population has no problem with size estimation.

### 3.2. Apparatus

**Figure 1:**
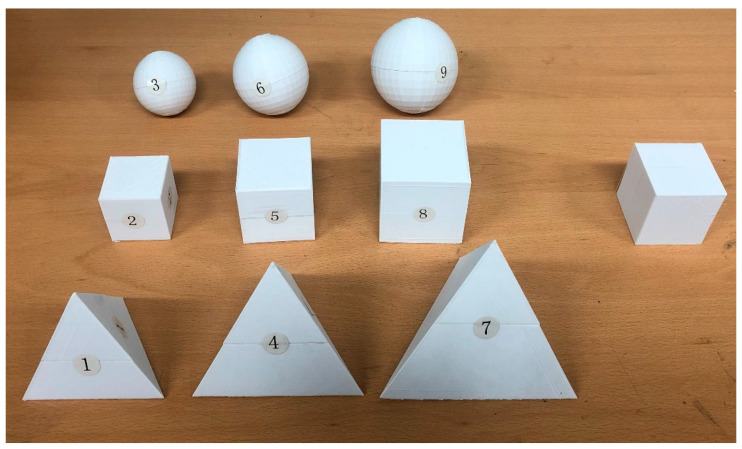
Nine objects and one reference object in the experiment (the reference object is the same as object 5).

The shapes of the objects used in this study are tetrahedron, cube, and sphere. They were made in three sizes corresponding to three levels of volume: 64,000 cm^3^, 125,000 cm^3^, and 216,000 cm^3^. The length of one side of the solid or diameter corresponding to these volumes is shown in Table 2. The weights of the objects were set to 100 g, 150 g, and 200 g. Note that the samples need to be a weight that a person can lift and must be of a size that can be held so that the person can feel the weight. Consequently, the factors of this experiment were 3 levels of volume and 3 levels of shape. Nine experimental conditions are shown in Table 3 and Figure 1.

### 3.3. Task

Participants actively lifted an object and performed a task of subjective rating indicators. Following the protocol of Ellis and Lederman [27], participants actively held and felt the object when lifting it. For reference, this method is known to increase the sensitivity of perceived weight evaluation [1]. When evaluating the weight of an object, the modulus method was used in which the experimenter assigned an appropriate number to a standard stimulus. Analysis of this method is simple compared to the modified modulus, in which the participant assigns an arbitrary number to a standard stimulus and evaluates the remaining experimental stimulus based on this, and the free modulus in which the experimental participant freely assigns the numerical value to the experimental stimulus without the standard stimulus [6]. After confirming the value of 100 for the standard intermediate size and weight (12,500 mm^3^, 150 g), participants were asked to lift the rest of the objects and rate the relative stimulus sizes. In addition, the reference object, which was not labeled in Figure 1, can be picked up and checked at any time

### 3.4. Procedure

Participants first familiarized themselves with the test protocol. In this process, the contents of the SWI phenomenon were not disclosed and the experimenter explained the purpose of the experiment not to be recognized as much as possible. The weight of the reference object was checked by participants first. As mentioned above, it is possible for participants to check at any time even when rating other objects. The evaluation order of the objects was determined according to the Latin square balancing. The experiment took about 30 min for each participant to evaluate all the objects. After the experiment, a predetermined participation fee was paid.

## 4. Results

### 4.1. Individual Traits

The effect of individual traits on the perceived weight was not significant, using ANOVA (Table 4). The perceived weight did not differ by individual. For example, gender did not have a significant effect on perceived weight (F(1,34) = 0.93, *p* = 0.34) and the interaction between gender and weight also had no significant effect on perceived weight (F(2,68) = 1.51, *p* = 0.23). Furthermore, the interaction between gender and shape was not significant (F(2,68) = 0.55, *p* = 0.58), nor was the three-way interaction of gender, weight, and shape (F(4,136) = 0.09, *p* = 0.99). The statistical results of the effect on the perceived weight of body weight and height were the same. If there was any interaction effect, the simple effect test was further applied [28].

### 4.2. Weight, Size, and Their Interaction

The effects of factors on the perceived weight were analyzed with repeated-measures ANOVA (Table 5). The effect of the weight (volume) of the object on the perceived weight was significant at a significance level of 0.05 (F(2,70) = 45.03, *p* < 0.0001) and the shape of the object was also significant (F(2,70) = 56.43, *p* < 0.0001). Additionally, the interaction effect between the object’s weight and shape was significant at a significance level of 0.05 (F(4,140) = 2.48, *p* = 0.046).

**Figure 2:**
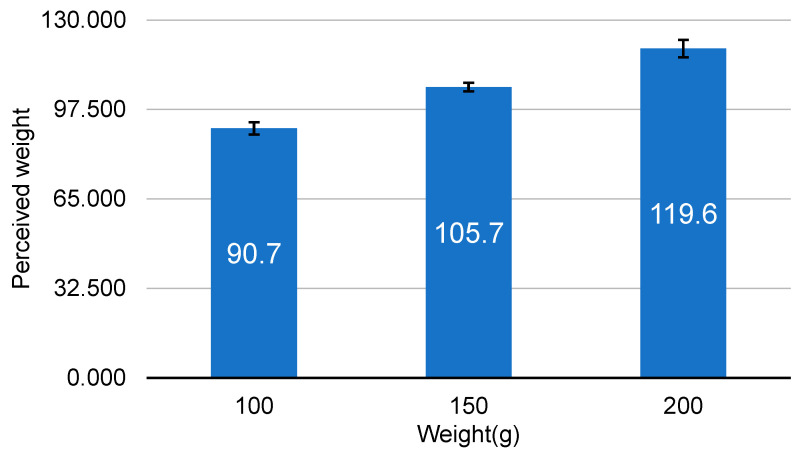
Perceived weight with 3 weights (volumes).

Due to the post hoc analysis of the perceived weight by the weight, the perceived weight increased significantly in direct proportion to the object’s actual weight (Figure 2). Bars represent standard errors. The perceived weights of the objects were all significantly different from each other in descending order of actual weight (*p* < 0.0001).

**Figure 3:**
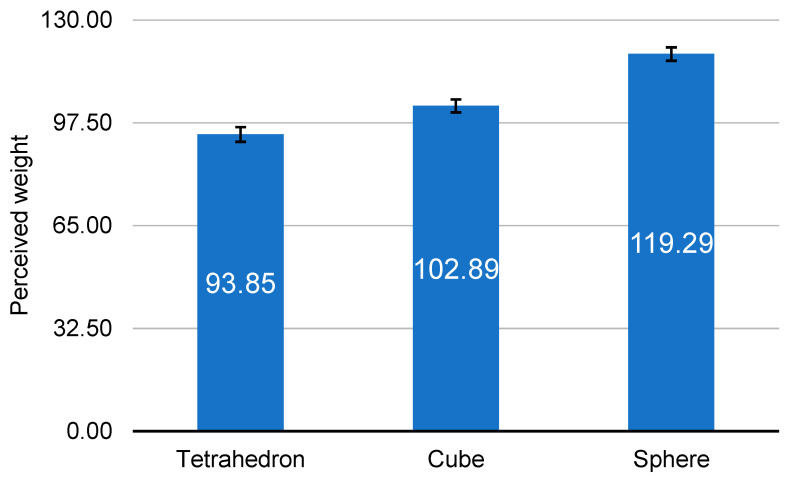
Perceived weight with 3 shapes.

In the post hoc analysis of perceived weight by shape, the object with the greatest weight was the sphere, followed by cube and tetrahedron (Figure 3). Bars represent standard errors. Their weights were statistically different (*p* = 0.001 for tetrahedron and cube, *p* < 0.0001 for the rest).

The interaction between the weight and the shape of the object is significant (*p* = 0.046). After applying simple effect tests of weights for each shape, all statistical differences in weight were significant (α = 0.05). The weights of 100, 150, and 200 g were all statistically different in a tetrahedron and the same was true in a cube and sphere.

**Figure 4:**
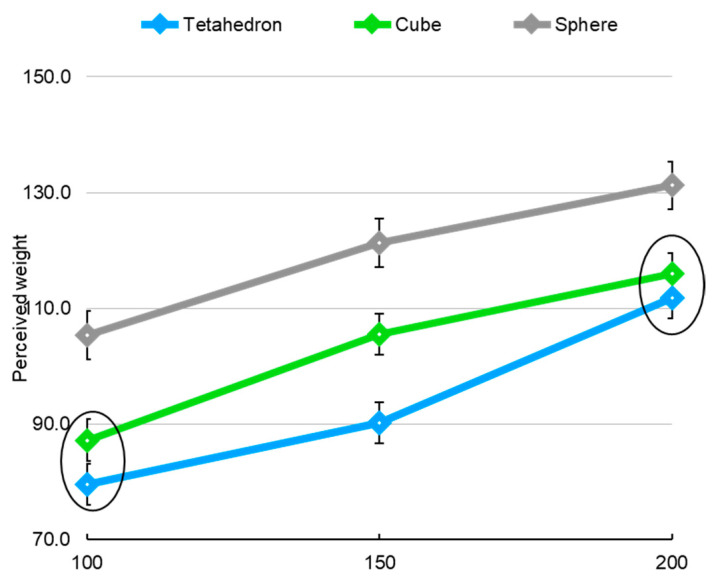
Interaction effects of weight and shape of objects on perceived weight (circles mean groups that did not differ statistically).

As a result of the simple effect analyses of the shapes at each weight, there was no difference in the perceived weight of the tetrahedron and the cube at 100 g and 200 g (*p* = 0.05 at 100 g, *p* = 0.25 at 200 g), but the weight of all shapes at 150 g was different (*p* < 0.0001 in all comparisons) (Figure 4). Bars represent standard errors.

## 5. Discussion

### 5.1. Shape Effect on Perceived Weight

This study experimentally showed that the shape of an object affects the perception of weight. In terms of the shape used, an existing study [15] only revealed that the weight of a tetrahedron is smaller than that of a cube when the weight (volume) is the same, but it does not show a statistical difference between a tetrahedron and a sphere and between a cube and a sphere. On the other hand, this study statistically shows that the weight of a tetrahedron is less than that of a cube and the weight of a cube is less than that of a sphere when the weight (volume) is the same, thereby demonstrating that the shape of an object affects the weight.

This study is significant for its analysis of the shape-weight illusion phenomenon, when object information was perceived using visual and tactile senses. Kahrimanovic et al. [15] analyzed the effect of shape on perceived weight using only the tactile sense without the visual sense. This study measured the perceived weight according to the shape in the bimodal perception situation, wherein the participants use both senses of sight and touch.

### 5.2. Weight-Estimation Model

This study contributed to proving that the SWI phenomenon is mediated by changes in the perception of the size of an object according to its shape. It is observed that the predicted difference in the values and the experimentally obtained measurement of the difference in weight between shapes are almost identical. Therefore, this study showed that the SWI phenomenon is mediated by size perception according to the shape of an object.

Kahrimanovic et al. [15] suggested a formula for predicting the perceived weight according to the size as follows: when the volume of a new object is doubled at the same weight, the perceived weight becomes 74% of the existing object and the weight decreases by 26%, where *W* is the perceived weight and *V* the perceived volume of objects. This formula, however, is applicable only when size information is tactilely transferred and it is difficult to apply to weight prediction when visual and tactile information is used.

(1) $$\frac{W_{new}}{W_{old}}={\left(\frac{V_{new}}{V_{old}}\right)}^{-0.43}$$

Kahrimanovic et al. [14] investigated the subjective sense of size in three solids (tetrahedron, cube, and sphere) with the same volume (Table 6). They reported that a tetrahedron was 32% larger than a sphere, a tetrahedron was 11% larger than a cube, and a cube was 21% larger than a sphere. Substituting this into the weight-based formula mentioned above, the perceived weight of a tetrahedron can be expected to be 18% lighter than a sphere, 5% lighter than a cube, and cube 11% lighter than a sphere. Note that a negative number means an object’s perceived weight is smaller than that of another object.

However, when both visual and tactile information were used in this study, the perceived weight of a tetrahedron was 27% lighter than a sphere, a tetrahedron was 10% lighter than a cube, and a cube was 16% lighter than a sphere. To compensate for the existing formula, if the exponential part is adjusted to −0.63 from −0.43, a tetrahedron is 28% lighter than a sphere, a tetrahedron is 8% lighter than a cube, and a cube is 16% lighter than a sphere. This is closer to the observed value than estimated by the existing formula. The weight prediction formula when both visual and tactile information must be modified is as follows:

(2) $$\frac{W_{new}}{W_{old}}={\left(\frac{V_{new}}{V_{old}}\right)}^{-0.63}$$

### 5.3. SWI as a Cognitive and Medical Phenomenon

The results obtained demonstrate a clear relationship between the perception of the weight of an object and its shape and size. These results may contribute to existing size-weight illusion studies supporting our findings. Study [29] showed that weight perception is influenced by a person’s expectations, as well as personal reactive and direct behavioral aspects. Research in [8] found that vision plays an important role in the assessment and subsequent correction of the applied load to the lifted object. Personal expectations were also an important factor in evaluating the applied lifting effort. Research in [30] proposed the hypothesis that the influence of object size on perceived weight is related to personal innate and phylogenetic aspects more than other conditions. Other results [31] showed that the size-weight illusion perception is related to personal features. For example, integrated human perception plays an important role together with a multimodal sensory environment.

Further, previous studies [19,22,23] showed the importance of human perception, including the weight and size of objects, as a diagnostic and monitoring tool for various health problems. This applies to violations of motor functions, cognitive, and coordination abilities of patients with neurological disorders and diseases (damages) of brain functions. The present study supports previous results and also expands them for use in health monitoring and recovery, as well as receiving therapeutic care after diseases, such as stroke, heart attack, Parkinson’s disease, and early developmental delay in children.

### 5.4. Research Limitations, Future Study, and Result Applications

Although this study demonstrated and explained the shape-weight illusion phenomenon, it still has some limitations. First, the weight levels used in this study were three levels of 100, 150, and 200 g, and the shape-weight illusion phenomenon in this study was verified at relatively lighter weights. Therefore, it is necessary to test whether this illusion occurs at heavier weights using different weights. Second, the geometry of studied objects was limited by tetrahedron, cube, and sphere shapes. To gain more information about the relationship between perceived weight and the shape of an object, the set of geometric shapes could be expanded to include, for example, a cylinder, a polygon, and various complex shapes in future studies. Third, there may be a limitation in the method of evaluating the perceived weight of samples. In this study, the evaluation was performed only once, even if the sample could be lifted several times. For example, participants can repeat the method of using two samples and comparing which one is heavier. Furthermore, the number of participants in this study was not as high as 40 and all participants were healthy and in their 20s. Based on the fact that our research can be applied for medical purposes in different age groups, in future studies, it will be necessary to expand the participant groups according to age (young children, adolescents, middle-aged people, the elderly) and health status (for example, healthy people, people who have had a heart attack, participants with Parkinson’s disease, children with intellectual disability).

The results obtained during the study can be applied theoretically and in practice. First, the findings broaden the knowledge base in human factors as well as the social sciences, with a focus on the perception of the surrounding world. From a practical point of view, the results can be used in medicine to develop new, as well as improve existing, diagnostic and rehabilitation methods for people with impaired motor functions and the ability to perceive the world around them. These problems can be congenital or acquired, including early childhood developmental problems, heart attacks, strokes, Parkinson’s disease, and paralysis. Further, the results can be applied in the field of entertainment for the development of new geometric puzzles with elements of illusions of perception. Moreover, the obtained findings can be used in the field of marketing and sales to develop more effective methods of product presentation and advertising in order to avoid misleading clients when choosing product packaging, which means increasing customer trust and loyalty to the brand/seller with replenishing the customer base. Despite satisfactory research results, further research has room for expansion and improvement in order to obtain more applicable results, especially in the field of medicine. In general, the presented research contains findings that can be applied in medicine, sociology, marketing, advertising, entertainment, and games. 

## 6. Conclusions

This study confirmed that the shape-weight illusion phenomenon is eventually caused by an object’s shape affecting perceived weight. Nine objects in a combination of three shapes (i.e., tetrahedron, cube, sphere) and three weights—volume being directly proportional to weight—were fabricated. In this experiment, 38 participants were asked to evaluate the weight of these objects by comparing them with the reference stimulus. The result showed that the shape of the object had a significant effect on its perceived weight. The perceived weight was the largest for a sphere, followed by a cube and a tetrahedron. Further, the perceived weight was the smallest for the tetrahedron and the greatest for the sphere. 

The results obtained can be applied in various fields, such as medicine, sociology, marketing, and entertainment. The findings can provide additional knowledge for medical researchers and experts to improve and develop recovery methods after neurological and brain diseases. The proposed research can supply specialists in the early development of children with new data to increase the effectiveness of applied methods for motor function monitoring. Moreover, experts in marketing, trade, advertising, and entertainment can find useful findings to improve their services. 

## Figures and Tables

**Table 1:** Previous research result.

Ref.	Hypothesis	Method	Result
[17]	Study of relationships between visual-haptic integration and early visual deprivation based on size-weight illusion	Experiments on the perception of weight and size of different objects depending on early visual deprivation experience	Size-weight illusion can be improved with adequate therapy for people with early visual problem experience
[18]	Proposal of the 3D virtual game for the development of the children’s fine motor skills	3D virtual experiment with different geometric and toy shapes and various difficulty task levels depending on personal children’s needs	An effective 3D environment for fine motor skill development was proposed with different geometric and toy objects and various difficult task levels
[19]	The connection between motor functions and cognitive abilities, including spatial thinking and deduction	1970 British Cohort Study (multi-disciplinary longitudinal monitoring with interviews)	The relationships between fine motor development, cognitive functions and spatial reasoning have been confirmed
[20]	Effectiveness of combination of acupuncture and occupation therapy for fine motor skills of children with cerebral palsy	Applying a new treatment based on a combination of acupuncture and occupational therapy among 80 cerebral palsy kids with fine motor skills issues	The proposed treatment method showed better performance for fine motor skills development in comparison with existing approaches among children with cerebral palsy
[21]	Study of occupational therapy effectiveness for fine motor function development. Connection of motor skills with self-care, mobility, and social function among preschool children	Long-term treatment of children using occupational therapy and observation of their ability of self-care, mobility, and social functions	The connection between fine motor skills, self-care function and mobility has been confirmed during the application of occupational therapy
[22]	Study of connection between visual-motor integration and executive functions among preschool children	Experiment with five tasks of copying different geometric shapes	The connection between manual dexterity, visual-motor integration, and executive functions was supported
[23]	Study of visuomotor response in Parkinson’s disease depending on the visuoperceptual function	Neurological outpatient evaluation of fourteen patients with Parkinson’s disease	Parkinsonian patients have medical issues with using sensory functions to perform the complex and new movements
[24]	Development of Bayesian model for perception of size and weight of different objects	The developed model of the perceived weight of objects is based on relations between object size and object density	The developed Bayesian model is able qualitatively and quantitatively evaluate and explain the size and weight illusion

**Table 2:** One side length of objects for three shapes (mm).

Shape	Small (64,000 mm^3^)	Medium (125,000 mm^3^)	Large (216,000 mm^3^)
**Tetrahedron**	82	102	122
**Cube**	40	50	60
**Sphere (diameter)**	49.6	62	74

**Table 3:** Experimental conditions.

Object No.	Volume	Weight (g)	Shape
1	Small	100	Tetrahedron
2	Small	100	Cube
3	Small	100	Sphere
4	Medium	150	Tetrahedron
5	Medium	150	Cube
6	Medium	150	Sphere
7	Large	200	Tetrahedron
8	Large	200	Cube
9	Large	200	Sphere

**Table 4:** Statistical significance of individual trait effects.

Factor	Subject Factor		
	Gender	Body Weight	Height
**Main effect**	F(1,34) = 0.93	F(4,31) = 0.74	F(3,32) = 0.48
	*p* = 0.34	*p* = 0.58	*p* = 0.70
**Subject factor × Weight**	F(2,68) = 1.51	F(8,62) = 1.52	F(6,64) = 2.06
	*p* = 0.23	*p* = 0.17	*p* = 0.7
**Subject factor × Shape**	F(2,68) = 0.55	F(8,62) = 1.58	F(6,64) = 1.34
	*p* = 0.58	*p* = 0.14	*p* = 0.25
**Subject factor × Weight × Shape**	F(4,136) = 0.09	F(16,124) = 1.04	F(12,128) = 0.15
	*p* = 0.99	*p* = 0.42	*p* = 0.99

**Table 5:** Effects of object weight (volume) and shape on perceived weight.

Source	SS	df	MS	F	*p*	η^2^
**Subject**	31,183.87	35	890.97			
**Weight**	45,247.93	2	22,623.96	45.03	<0.0001	0.233
**Weight × Subject**	35,170.08	70	502.43			
**Shape**	35,920.46	2	17,960.23	56.43	<0.0001	0.185
**Shape × Subject**	22,278.65	70	318.27			
**Weight × Shape**	1643.00	4	410.75	2.48	0.046	0.008
**Weight × Shape × Subject**	23,156.91	140	165.41			

**Table 6:** Magnitude of the bias between three shapes (%).

Condition	Type of the Illusion			
	Shape-Size (Kahrimanovic et al., 2010)	Shape-Weight (Expected)	Shape-Weight (Observed)	Shape-Weight (Calibrated) −0.63
**Tetra-Sphere**	32	−18	−27	−28
**Tetra-Cube**	11	−5	−10	−8
**Cube-Sphere**	21	−11	−16	−16

## Data Availability

Not applicable.
